# Editorial: Infectious disease control in the microbial functional genomics era

**DOI:** 10.3389/fmicb.2026.1934262

**Published:** 2026-08-25

**Authors:** Muhammad Yasir, John D. Klena, Moataz Abd El Ghany

**Affiliations:** 1 Quadram Institute, Norwich, United Kingdom; 2 Centre for Microbial Interaction, Norwich, United Kingdom; 3 Division of Global Health Protection, United States Centers for Disease Control and Prevention, Atlanta, GA, United States; 4 The University of Sydney School of Public Health, The University of Sydney, Sydney, NSW, Australia

**Keywords:** antimicrobial resistance, functional genomics, metagenomic diagnostics, mobile genetic elements, novel therapeutics, phage therapy

Functional genomics is fundamentally important for our understanding of infectious diseases. While high-throughput sequencing technologies have enabled unprecedented characterization of microbial pathogens, a critical gap persists between the vast accumulation of sequence data and our comprehension of gene function. With microbial sequence databases doubling every 2 years, the challenge is no longer generating genomic data but rather deciphering the functional significance of millions of uncharacterized genes. This Research Topic brings together 15 contributions that demonstrate how functional genomics approaches are bridging this knowledge gap and providing actionable insights for infectious disease control.

The transition from descriptive genomics to functional understanding represents a paradigm shift in microbiology. Traditional whole-genome sequencing (WGS) excels at tracking transmission dynamics, identifying resistance determinants, and characterizing outbreak strains. However, these approaches cannot predict which genes are essential for bacterial survival under specific stress conditions, which metabolic pathways enable persistence in host environments, or how bacteria adapt to antimicrobial challenges. The articles in this Research Topic showcase the power of functional genomics technologies—including transposon insertion sequencing (TIS), transcriptomics, proteomics, and genome-wide association studies, to understand these critical questions.

Several contributions exemplify the impact of high-resolution functional genomics on understanding bacterial stress responses and antimicrobial resistance mechanisms. Yasir et al. employed TraDIS-Xpress to reveal fundamentally different genetic requirements for *Escherichia coli* survival under immediate heat shock vs. stepwise adaptation. Their genome wide screen identified 530 genes contributing to heat stress survival, with striking differences between shock and adaptation strategies. Under immediate heat shock, cells survived through cellular simplification, including potential cell wall loss, while stepwise adaptation enabled energy intensive protective responses maintaining normal cellular architecture. This work demonstrates how prior environmental exposure dramatically reshapes the landscape of viable survival strategies, with implications for understanding bacterial persistence in food processing and clinical environments.

The topics focus on antimicrobial resistance reflects the urgent global health challenge posed by multidrug-resistant pathogens. Multiple studies employed whole-genome sequencing to characterize resistance mechanisms in priority pathogens designated by the World Health Organization. Liu et al. analyzed *Klebsiella pneumoniae* isolates from cancer patients, revealing considerable diversity in resistance genes, virulence factors, and sequence types, with different clonal lineages predominating across tumor types. Similarly, comprehensive genomic profiling of cefotaxime resistant *Haemophilus influenzae* from Scandinavia (Skaare et al.) and methicillin resistant *Staphylococcus aureus* from Saudi Arabia (Alhejaili et al.) documented the extensive expansion of virulent multidrug resistant international clones, highlighting the critical role of genomic surveillance in tracking resistance dissemination.

Several contributions examined the mobile genetic elements and evolutionary mechanisms driving resistance emergence. Medugu et al. investigated multidrug-resistant *E. coli* from Nigeria, revealing virulence-mobile element linkages and phylogenetic diversity that inform our understanding of resistance spread in resource-limited settings. Dong et al. characterized rare NDM-1-producing carbapenem-resistant *Acinetobacter baumannii* isolates, providing insights into the genetic contexts enabling the emergence of novel resistance combinations. Teng et al. demonstrated how single mutations can mediate dramatic virulence changes within individual patients, tracking *wzc* mutations altering K1-type *K. pneumoniae* pathogenicity during infection progression.

The application of functional genomics to identify novel therapeutic targets represents a major theme across multiple articles. Santos et al. explored the diverse virulence potential of atypical enteropathogenic *E. coli*, investigating whether hybrid pathotypes are emerging that combine virulence factors from multiple lineages. Zhu et al. characterized a fumarate reductase mutant of *Salmonella* enteritidis, assessing its immune-protective efficacy and demonstrating how functional genomics can guide vaccine development. Shaw et al. revealed that dietary oligosaccharides alter cellular respiration and amino acid metabolism in *Salmonella* spp. through epithelial cell association, providing insights into nutrition-pathogen interactions.

Prophage encoded antimicrobials offer promising alternatives to conventional antibiotics. Raposo et al. conducted comprehensive genomic analysis of prophages from 158 *A. baumannii* genomes, identifying 166 genes encoding putative lysins with potential therapeutic applications. Their phylogenetic analyses revealed remarkable prophage diversity and local clustering, highlighting the abundance of prophage-encoded antimicrobials in this critical priority pathogen. This work exemplifies how mining bacterial genomes for prophage elements can accelerate discovery of novel therapeutics.

The diagnostic revolution enabled by metagenomic next-generation sequencing (mNGS) is showcased in two complementary studies. Chen et al. and Li et al. independently evaluated mNGS for pathogen detection in suspected pulmonary infections, demonstrating significantly higher detection rates (86.17% vs. 67.55%) compared to conventional microbiological tests. These studies identified diverse pathogens including bacteria, fungi, viruses, and atypical organisms often missed by culture-based methods. The effectiveness of antibiotic treatment decisions based on mNGS results (40.60%) and improved outcomes for infections caused by difficult-to-culture pathogens underscore the clinical utility of unbiased sequencing approaches.

Technological advances continue expanding the functional genomics toolkit. Ivanova et al. assessed sample multiplexing impacts on detection of bacteria and antimicrobial resistance genes in pig microbiomes using long-read sequencing, addressing practical considerations for large-scale surveillance programs. Hou et al. demonstrated nanopore sequencing applications for HPV integration profiling, extending functional genomics approaches to viral oncogenesis and cervical precancerous lesions.

Looking forward, several research priorities emerge from this Research Topic. First, integrating functional genomics data with traditional epidemiological and clinical information will maximize impact on infection control practices. Second, expanding functional screens to diverse bacterial species, particularly fastidious organisms, will broaden our understanding of microbial physiology. Third, developing predictive models that incorporate functional genomics data to forecast resistance emergence, outbreak dynamics, and treatment outcomes represents an important frontier. Fourth, translating functional insights into tangible therapeutic interventions, whether novel drug targets, live-attenuated vaccines, or phage-derived antimicrobials, remains a high-priority goal.

The convergence of high-throughput sequencing, powerful bioinformatics, and sophisticated experimental genetic techniques applied to pathogens has positioned functional genomics at the forefront of infectious disease research. This Research Topic demonstrates how moving beyond descriptive genomics to functional understanding enables identification of essential genes, characterization of adaptive mechanisms, discovery of therapeutic targets, and development of precision diagnostic approaches. As antimicrobial resistance continues threatening global health security, functional genomics provides indispensable tools for understanding and combating bacterial pathogens. The coming decade would be an opportunity to close the gap between knowing a pathogen's genome and understanding how it causes disease, and the work presented here paves a clear path toward that goal.

